# Responses of two *Acacia* species to drought suggest different water-use strategies, reflecting their topographic distribution

**DOI:** 10.3389/fpls.2023.1154223

**Published:** 2023-06-05

**Authors:** Daphna Uni, Efrat Sheffer, Tamir Klein, Rachamim Shem-Tov, Nitzan Segev, Gidon Winters

**Affiliations:** ^1^ Institute of Plant Sciences and Genetics in Agriculture, The Robert H. Smith Faculty of Agriculture, Food and Environment, The Hebrew University of Jerusalem, Rehovot, Israel; ^2^ Department of Plant and Environmental Sciences, Weizmann Institute of Science, Rehovot, Israel; ^3^ Acacia Research Center, The Dead Sea-Arava Science Center, Masada, Israel; ^4^ Department of Life Sciences, Ben-Gurion University of the Negev, Eilat, Israel

**Keywords:** drought, desert, *Acacia* trees, plantarray, transpiration

## Abstract

**Introduction:**

Soil water availability is a key factor in the growth of trees. In arid deserts, tree growth is limited by very dry soil and atmosphere conditions. *Acacia* tree species are distributed in the most arid deserts of the globe, therefore they are well adapted to heat and long droughts. Understanding why some plants do better than others in some environments is a key question in plant science.

**Methods:**

Here we conducted a greenhouse experiment to continuously and simultaneously track the whole-plant water-balance of two desert *Acacia* species, in order to unravel their physiological responses to low water availability.

**Results:**

We found that even under volumetric water content (VWC) of 5-9% in the soil, both species maintained 25% of the control plants, with a peak of canopy activity at noon. Moreover, plants exposed to the low water availability treatment continued growing in this period. *A. tortilis* applied a more opportunistic strategy than *A. raddiana*, and showed stomatal responses at a lower VWC (9.8% *vs*. 13.1%, t_4_= -4.23, p = 0.006), 2.2-fold higher growth, and faster recovery from drought stress.

**Discussion:**

Although the experiment was done in milder VPD (~3 kPa) compared to the natural conditions in the field (~5 kPa), the different physiological responses to drought between the two species might explain their different topographic distributions. *A. tortilis* is more abundant in elevated locations with larger fluctuations in water availability while *A. raddiana* is more abundant in the main channels with higher and less fluctuating water availability. This work shows a unique and non-trivial water-spending strategy in two Acacia species adapted to hyper-arid conditions.

## Introduction

Arid and semi-arid areas cover up to 45% of the Earth’s land surface (Bastin et al., 2017). Although desert habitats are poor in water and nutrients, Acacia trees inhabit many deserts around the globe (Maslin et al., 2003), such as the Sahara (Essendoubi et al., 2007), the Arabian desert (Seleem et al., 2013) and the Negev desert of Israel (Halevy and Orshan, 1972; Ross, 1981; Danin, 1983). In these arid habitats, Acacias grow primarily in the main channels of ephemeral streams (i.e. “wadis”) (Munzbergova and Ward, 2002; Isaacson et al., 2017; Armoza-Zvuloni et al., 2022), tolerating extreme conditions of high radiation, temperatures and vapor pressure deficit (VPD), with infrequent and short rain events (Goldreich and Karni, 2001; Uni et al., 2022). However, in this dry wadi system, short but intense rain events create flash floods (approximately twice a year) entailing rapid and high fluxes of water that arrive fast and disappear fast (Dahan et al., 2007; Dayan et al., 2021). The stream-beds of these wadis are composed mostly of permeable, coarse alluvial sediments that promote rapid infiltration of floodwater into deep soil layers, resulting in low availability of water in the upper 5-7 m of the soil (Dahan et al., 2007; Winters et al., 2015).

Soil water availability is a key factor in the growth and development of trees (Klein et al., 2015; Reich et al., 2018), as it is the buffer linking precipitation and tree water usage (Veihmeyer and Hendrickson, 1950). Eckes-Shephard et al. (2021) studied the direct response of tree growth to soil water (in the Swiss Alps) and found in their model that 60% of tree ring widths were explained by volumetric soil water content. The importance of soil water availability is especially strong in semi-arid and arid environments (Noy-Meir, 1973; Reynolds et al., 2004; Albrecht et al., 2014), where the combination of low precipitation with dry atmosphere results in a high water demand by the trees (Chaves et al., 2003). An example of this can be seen in the semi-arid pine forest in Israel (the southern edge of pine forests in the world), where Klein et al. (2014) found that a reduction of only 5% in VWC caused a reduction of 14-34% in tree growth.

In response to limited soil water content, trees are able to partially control their water loss by regulating stomatal conductance (Kirkham, 2014). Regulation of stomatal aperture is suggested to be especially important in semi-arid and arid environments, mainly during dry periods (Eamus and Prior, 2001; Klein et al., 2011; Klein et al., 2014). However, unlike most studied dryland trees, desert Acacias were found to show relatively low stomatal regulation (closure) for the prevention of stomatal water loss (Do et al., 2008; Winters et al., 2018). For example, *Acacia tortilis* in the northern Sahel (one of the driest savannahs in the world) showed consistent patterns of water loss throughout the year with stable diurnal sap flow rates (29 ± 4.4 L per day), despite significant seasonal fluctuations in the environmental conditions (Do et al., 2008). Another example from *A. tortilis* trees in the hyper-arid desert of Israel includes work by Winters et al. (2018) that showed relatively low seasonal variability in the rate of diurnal sap flow (12.5 ± 3 L per day) even though relative humidity ranged from 4-94% throughout the year. Furthermore, in contrast to most studied desert trees, the highest CO_2_ assimilation and transpiration rates of these same Acacias were surprisingly during midday in the summer, when temperatures and VPD were at their maximum (Uni et al., 2022). However, determining whether low soil water availability takes part in stomatal regulation of Acacia trees is difficult when based only on field observations since the trees in the field might have deep and wide root systems (Ludwig et al., 2003; Do et al., 2008; Sher et al., 2010) which can provide them with a constant water supply throughout the dry season.

Controlled experiments conducted on Acacia seedlings also suggest a surprisingly limited capacity to regulate water loss. For example, in a controlled drought experiment, in which irrigation was withheld, no differences in hydraulic conductance of water-stressed and non-stressed trees were found (Otieno et al., 2005). Cory et al. (2022) found that *A. tortilis* seedlings tend to take an anisohydric ‘water-spender’ (i.e., “risk-taking”; (Sade et al., 2012)) strategy, particularly at moderate levels of drought. However, these experiments were done with seeds collected from populations adapted to much wetter environments (900-1000 mm year^-1^) compared to populations that come from arid and hyper-arid deserts (20-70 mm year^-1^) as described in this study (see below). Information on the specific strategy that desert Acacias use in order to survive and grow in arid and hyper-arid environments is still missing, especially, on the response of canopy conductance dynamics to low soil water availability.

In this study, we aim to answer the question- what are the continuous physiological responses (canopy conductance and biomass accumulation) of two dominant desert Acacias keystone species to limited soil water availability? Moreover, we asked whether these physiological responses facilitate their survival and establishment during early life stages in a harsh desert environment. To shed light on these questions, we tracked the whole-plant water-balance regulation traits under well-watered and water-limited conditions in a continuous and simultaneous experiment on an array of lysimeters (Halperin et al., 2017; Dalal et al., 2020; Jaramillo Roman et al., 2021). By controlling the soil water content, gradually reduced from 17% (full saturation) to 5% (drought), while all other environmental conditions (light, temperature, soil type, seedlings age, and root depth) were kept the same for all plants, we were able to isolate the dynamics of stomatal conductance and biomass gain response to a single parameter - soil water content. This controlled experiment is a follow-up study to a two-year field study on mature trees of the same species, growing natively under hyper-arid conditions (Uni et al., 2022). Our findings point to a surprising strategy applied by desert Acacias under low water availability conditions.

## Materials and methods

### Plant species and treatments

**Figure 1:**
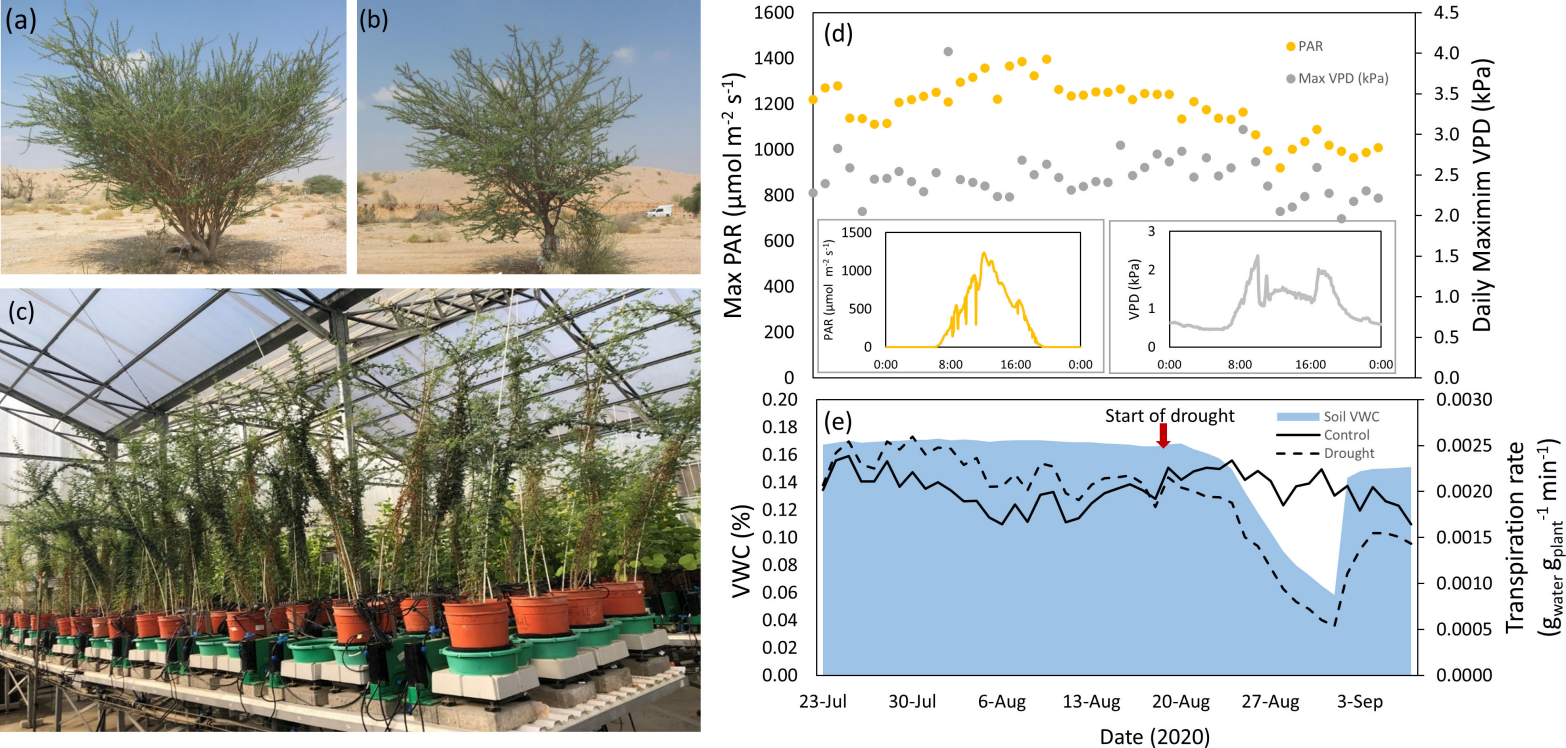
*Acacia tortilis* **(A)** and *A. raddiana* **(B)** trees in their natural habitat (hyper-arid desert), seedlings of the same population growing in the greenhouse on the lysimeters system (plant array - a high-throughput, multi-sensor physiological phenotyping gravimetric platform) **(C)**. The abiotic conditions in the greenhouse during the experiment (July-September 2020): daily maximum photosynthetic active radiation (PAR) and vapour-pressure deficit (VPD) with the daily cycles of these parameters on a diurnal scale (representative days in insert) **(D)** alongside average soil volumetric water content (%) in the drought pots, and plants transpiration rate in the two water treatments (dashed line for drought treatment, non-dashed line for control treatment) **(E)**.

Seeds of *Acacia tortilis* (**Figure 1A**) and *A. raddiana* (**Figure 1B**) were collected from a wild population (10 trees, 5-20 m distance among them) in the Arava valley at the Negev Desert of Israel (Wadi Sheizaf, 30.721222’N, 35.268366’E; elevation -137 m a.s.l) during July 2019 and stored according to Israel’s Plant Gene Bank protocols (https://igb.agri.gov.il/web/index.php). In December 2020 we chipped the hard seed-coat and germinated the seeds in agar plates in an incubator (12:12 light:dark, 20°C), according to protocols developed for Acacia species (Tran et al., 2018). Germinating the seeds by clipping the seed’s seminal cortex mimic animal herbivory or erosion in a flash flood, the most common natural ways for Acacia seeds to germinate (Polak et al., 2014). After germination, we transplanted the seedlings into small pots (6.5 ×6.5×7.8 cm) and grew them in a controlled temperature glass greenhouse (28˚C/22˚C day/night under natural light conditions. All seedlings were given uniform optimal conditions of full irrigation (4 drippers irrigating until pot saturation) with addition of nutrients (poly feed N:P:K 17:10:27, Haifa Chemicals, Haifa, Israel). Six months after the seedlings were sown, they were transplanted into 4 L pots (20.0 × 15.5 × 16.5 cm) filled with uniform grain size sand (0.6–1.0 mm) with one plant per pot, and transferred to a semi-controlled experimental greenhouse (at the iCORE Center for Functional Phenotyping, Rehovot, Israel) for the rest of the experiment (https://plantscience.agri.huji.ac.il/icore-center). This experimental platform enables researchers to follow the physiological behavior of a large number of plants, simultaneously, and with very high time resolution, in response to a decrease in water availability. The water parameters measured here are based on the pot weight, therefore, they represent the actual water used by the plants, a parameter which is not possible to measure in the field. This platform has gained widespread use in numerous studies (Halperin et al., 2017; Fox et al., 2018; Dalal et al., 2020; Jaramillo Roman et al., 2021; Houminer et al., 2022), including a specific correlation between “classic” measurement of stomatal conductance (promoter and gas analyzer) to the system measurements (R^2 = ^0.9914) (Jaramillo Roman et al., 2021).

### Lysimeters - experimental design and data collection

The experiment was conducted in the summer between July and September 2020 when annual radiation, temperature and VPD are the highest (**Figure 1D**). It should be noted that the highest VPD measured in the greenhouse was only 3kPa, which occurs in the winter season in the Acacia’s natural habitat (Uni et al., 2022). Therefore, the comparison with the conditions in the field is limited. It also should be noted that reaching very high VPD in greenhouses is almost impossible (Shamshiri et al., 2018). The pots with the seedlings (16 per species, 8 per treatment, the total number of seedlings*=*32) were placed on a weighing lysimeter system, one pot per scale. The different pots were placed on the experimental table in a randomized block design determined by a randomizing software in order to minimize environmental and edge effects (Dalal et al., 2020) (**Figure 1C**). Initial calibration for each weigh-scale was performed at the beginning of the experiment on all lysimeters under constant load weights (1 kg and 5 kg) using the Plantarray auto-calibration application (Halperin et al., 2017; Dalal et al., 2020).

The set-up of the experiment was comprised of highly sensitive weighing lysimeters, and each pot was weighed and its irrigation controlled individually by a separate control unit, which also collected continuous data. Fertilizer (poly feed N-P-K 5:10:27, Haifa Chemicals, Haifa, Israel) was supplied to the plants through the irrigation system (fertigation). The pots fit tightly in the system containers, and the containers have several drainage holes at different heights which enabled drainage of excess water according to the irrigation regime. To prevent evaporation from the soil surface of the pot, circular PVC covers were placed on the surface of the pots, tightly surrounding the plant stem. For the full irrigation treatment, fertigation was applied as 15 minute pulses every 2 hours, throughout the night, allowing optimal water dispersal and drainage in the pot, ensuring that we reach the maximal pot capacity. VWC and atmospheric conditions (temperature, radiation, VPD) were simultaneously monitored at several locations around the experimental table as described in Dalal et al. (2020).

### Drought and recovery treatments

The continuous physiological experiment was designed to monitor the response of Acacia seedlings to a declining soil water content (SWC). The experiment was carried out for a total of 47 days. After an establishment period of 26 days during which the soil of all pots was irrigated every day to full saturation (16.7% in our sandy soils) the pots were divided into two groups - drought and control. For the drought group only, irrigation was decreased gradually for each individual pot until reaching a VWC of 5% after 14 days which was considered extreme drought, based on soil measurements at the summer period in the field (data not shown). In order to ensure that all the drought treatment plants were exposed to a uniform drought treatment, the deficit irrigation regime that was applied to each pot, was based on the previous day’s transpiration of that same pot, by the system’s feedback-irrigation controller, which supplied each pot with only 20% of the previous day’s transpiration demand (Dalal et al., 2020). The daily transpiration rates and the VWC were closely monitored throughout the drought treatment. After 14 days of drought treatment when the plants’ transpiration rates were minimal, full irrigation was restored for a recovery periord, simulating a flashflood which brings high amounts of water to the wadis in the desert (**Figures 1D, E**).

### Measurements of plant physiological traits

The kinetics of plant-water-relations (recorded by the system every 3 min.) and quantitative physiological traits of the plants were determined simultaneously for all plants, following Halperin et al. (2017) with minor modifications, and included the following parameters: daily transpiration (g_water_/day/plant), transpiration rate (E, g_water_/g_plant_/min), whole-canopy stomatal conductance, divided by VPD (g_sc_, g_water_/g_plant_/min)), and plant water-use efficiency (daily biomass gain/daily transpiration). Daily biomass gain was calculated by the system each morning at 04:00 am, after the end of irrigation and after full drainage was achieved (Halperin et al., 2017) by subtracting the overall weight of the pot from the weight measured on the previous day. The daily percentage of growth was calculated by dividing daily biomass gain (g) by the final plant weight at the end of the experiment minus the initial plant weight. To avoid bias of calculating the growth rate we used the relative growth rate (RGR) equation as described in Hoffmann and Poorter (2002). The RGR equation is based on dry mass, while the lysimeters system is measuring the fresh weight of the seedlings, therefore we calculated also the fraction of dry mass from fresh mass (%) at the last day of the experiment (average of 32%) and used it as a calibration value. VWC was measured directly using soil sensors (Decagon, 5TE, USA). The point at which transpiration rate began to be affected by limited soil water availability was determined by the piecewise linear fit of the transpiration rate and VWC of the plants subjected to the drought treatment.

### Osmolality measurement

From each treatment, leaves were sampled at three time points (5 days before drought, at the peak of drought, and 4 days after the start of recovering) and placed in 1.5 ml plastic tubes that were immediately frozen in liquid nitrogen (*n=*5 for each treatment, at each time point). The samples were stored in -80°C until extraction. To extract the sap, the tubes were centrifuged for 3 min. at 13,000 RPM (Eppendorf 5424R, UK. The extracted sap was kept on ice (max. 60 min.) until measured in a vapor pressure osmometer (Vapro 5600; Wescor Inc., USA) and the mean of two technical repetitions was taken as leaf osmolality (mmol kg^−1^).

### Species distribution maps in the ephemeral rivers

The stream system of any drainage basin can be quantitatively expressed in terms of stream order; 1^st^ order wadis are defined as the smallest channel that flow toward 2^nd^ order channels and so on to the highest number of order, which drainage all the water in a certain catchment area, the main channel. the small channels (outside the main one) are characterized by fast water movement toward the main channel (Horton, 1945; Lange, 2005). Thus, most of the water (and sediments) accumulate in the main channel (Levick et al., 2008; Stavi et al., 2015). Based on a standard protocol to monitor Acacia trees in the Arava Desert (Groner et al., 2017) plots of 1 hectare were defined in three wadis (ephemeral rivers) (Stavi et al., 2015). In each plot we measured and recorded the location (using GPS) and species identity of each tree (*A. raddiana* or *A. tortilis*) and the relative location within the wadi (wadi order). This information was used to create the distribution maps in the wadi and calculate the relative abundance of each of the species as the number of individuals of each species from the total number of Acacia trees observed at each monitored plot.

### Statistical analysis

We monitored the diurnal dynamics of the canopy conductance in both species before and during drought, and after drought recovery. These continuous data of the weight of each pot from the lysimeter system were filtered and summarized using the SPAC (Soil-Plant-Atmosphere-Continuum) analytic software embedded in the Plantarraysystem (PlantDitech, Yavne, Israel). To test how canopy conductance was influenced by low water availability we tested the reaction of the canopy conductance values during noon (10:00-14:00) to the gradual reduction in irrigation (2 species, 47 days, drought and control treatments, 5-7 replicates per treatment). We also correlated daily growth and VWC of the pot to examine differences between the control and drought treatments in both species. All statistical analyses (ANOVA between four groups; two species and two water treatments, or t-test comparing two species under drought treatment only) were performed using the JMP^®^ 15.0 Pro statistical package (SAS Institute, Cary, NC, USA) unless otherwise specified. Box plots and continuous line graphs were generated using R software and the interface R Studio (R Development Core Team 2006, 1.2.5033 (R Core Team, 2013)).

## Results

### Overall water balance under drought

The Acacia seedlings in the greenhouse (**Figure 1C**) were exposed to uniform conditions of light, temperature and air humidity, entailing also similar VPDs (**Figure 1D**). It should be noted that the conditions in the greenhouse were only semi-controlled, thus dictated by the outside conditions with maximum temperatures limited to 35 ˚C by the greenhouse cooling system. To test the response of the Acacias to limited water availability under the hottest conditions, we conducted the experiment during the summer months at the highest temperatures of the year (which ranged from 22- 33 ˚C in the greenhouse). The daily maximum values of VPD in the greenhouse throughout the experiment ranged from 2.5-3 kPa, and the average PAR light was 1100 µmol photons m^-2^ s^-1^ (**Figure 1D**). At the time of extreme drought (after 14 days of decreasing irrigation inputs), transpiration rate was lower in drought-subjected plants (from both species) by 75% as compared to the well-watered control plants (0.00052 g_water_ g_plant_^-1^ min^-1^; 0.0019g_water_ g_plant_^-1^ min^-1^, respectively, F_1,46 = _1.67, *p* = 0.04) (**Figure 1E**). Both species maintained approximately 25% of their canopy conductance even at the very low VWC of 5%, enabling photosynthetic activity under extreme drought conditions.

**Figure 2:**
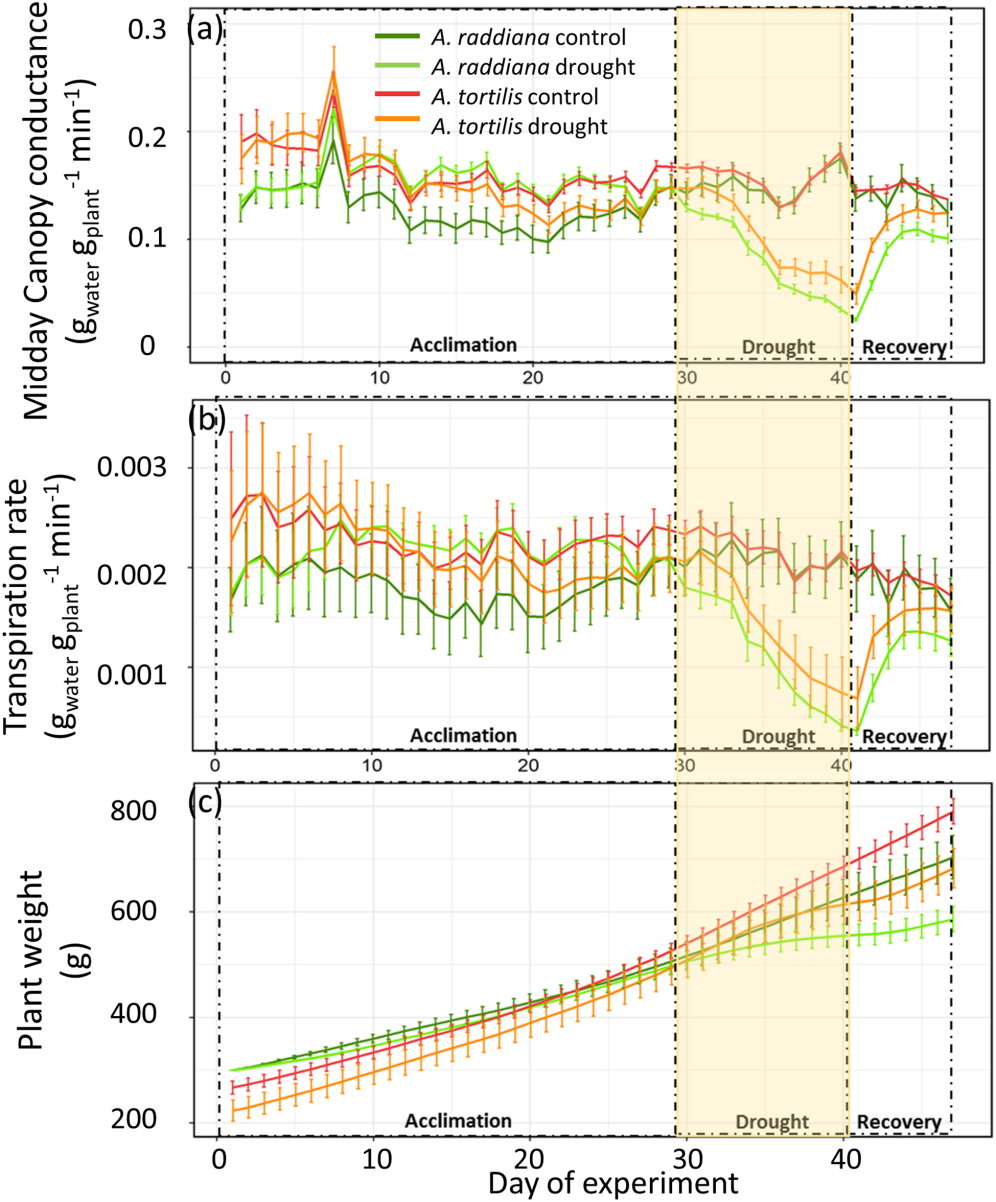
Midday canopy conductance (g_water_ g_plant_^-1^ min^-1^_)_ **(A)**, transpiration (g_water_ g_plant_^-1^ min^-1^_)_ **(B)**, and plant wet weight (g) **(C)** measured throughout the experiment. Shown are averages (n=7-5) of *Acacia tortilis* and *A. raddiana* seedlings exposed to drought and control during the acclimation period (23 days), followed by a 14 days of gradual drought exposure, followed by one week of recovery.

Although midday canopy conductance (g_sc_) of both Acacia species was significantly reduced by the drought treatment as compared to their well irrigated controls, *A. tortilis* maintained higher g_sc_ than *A. raddiana* at the peak of drought (VWC 5-9%) (Average ± SE values of 0.05 ± 0.021, 0.02 ± 0.006 g_water_ g_plant_^-1^ min^-1^, respectively, F_1,7 = _26.24, *p* = 0.002) (**Figure 2A**, yellow frame). In addition, during the recovery phase, *A. tortilis* seedlings restored more than 80% of their initial unstressed levels of g_sc_ and transpiration rate after only 5 days, while in *A. raddiana* g_sc_ and transpiration rates were not fully restored for the remaining duration of the experiment (**Figures 2A, B**).

Biomass gain for the seedlings at the end the experiment ranged from 500 to 800 g (47 days), with no significant difference among the control and the drought treated plants within each species (**Figure 2C**). However, although *A. tortilis* were initially smaller than *A. raddiana* at the start of the experiments (**Figure 2C**), they gained 16% more biomass than *A. raddiana* in both control and drought treatments.

### Diurnal patterns of *Acacia* sp. under different VWC

**Figure 3:**
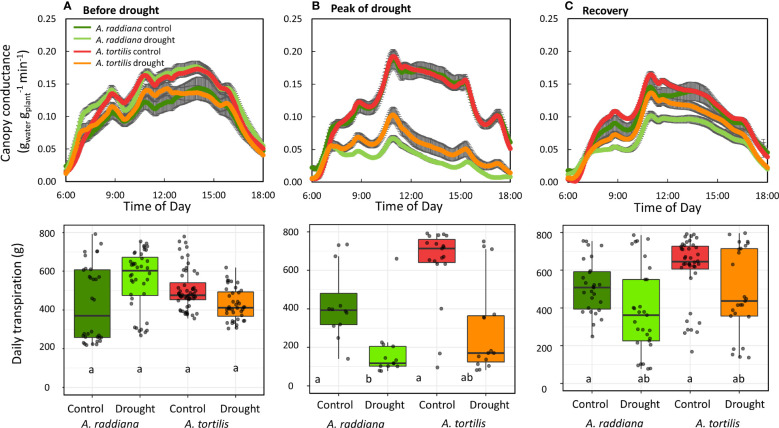
Diurnal pattern of canopy conductance (g_sc_) **(A)** before drought, **(B)** at the peak of drought, and **(C)** at the first day of recovery. Error bars represent SE, boxplots represent the total daily transpiration of each group (n=5-7), first to third quantiles; the middle line represents the median. The values of g_sc_ are means of 7 days before drought, 3 days in the peak of drought and all the recovery period.

We monitored the diurnal dynamics of the canopy conductance in both species before, during, and after drought (**Figure 3**). The overall diurnal g_sc_ patterns were similar in both species and were not affected by drought. From first light, the g_sc_ increased throughout the morning reaching its first peak at ~9:00 am, followed by a small reduction (due to a decline in VPD in the greenhouse), and then stabilized at maximum rates for several hours (between 11:00 and 15:00) followed by a reduction from 15:30 until dark. This general diurnal hourly trend was maintained throughout the experiment, even under extreme drought (**Figure 3B**) and throughout the recovery phase (**Figure 3C**) with only amplitude changing. Under well-watered conditions preceding the drought treatment, whole plant daily transpiration of the seedlings of all four groups (species and treatments) ranged from 430 to 700 g of water per day with no significant differences among the two species (**Figure 3A**). Reduction in water availability (drought treatment) resulted in a decline of seedling transpiration rates for both species compared to their controls (**Figure 3B**). The transpiration rate for *A. tortilis* was reduced by 47% compared to its control after 14 days of extreme drought, while for *A. raddiana* under the same conditions, transpiration rate was reduced by 68%. Upon recovery (**Figure 3C**), the g_sc_ of *A. tortilis* plants was quickly restored, reaching values of 82% of the control seedlings by the end of the experiment, while g_sc_ of *A. raddiana* reached only 70% of original values even after 7 days of full irrigation.

**Figure 4:**
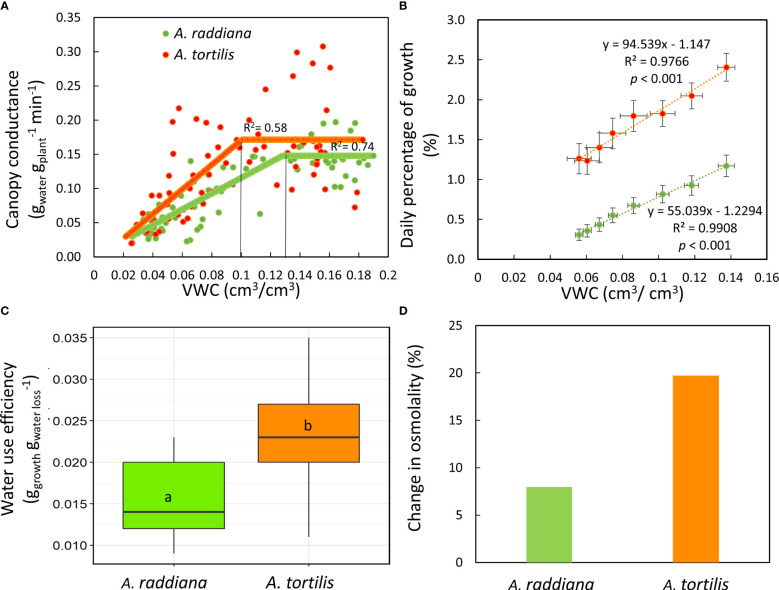
Comparisons of canopy conductance **(A)** and daily growth **(B)** responses to soil volumetric water content (VWC %) in *A*. *tortilis* (orange) *and A. raddiana* (green) seedlings under drought treatment. Significant differences in whole plant water use efficiency **(C)** and the percentage of change from control in leaf osmolality **(D)**.

To better understand the specific response of the two species to the imposed drought, we analyzed how canopy conductance and daily growth responded to the availability of water in the soil (VWC) under the drought treatment (**Figures 4A, B**). For *A. raddiana* a reduction in g_sc_ was first apparent when VWC reached 13.1%, while the g_sc_ of *A. tortilis* began declining only at VWC of 9.8% (**Figure 4A**), maintaining high g_sc_ values for a longer period under lower VWC values. This gap allowed *A. tortilis* seedlings to gain more biomass (**Figure 4B**) under more extreme water limitations, resulting in 2.2 times higher biomass gain (growth) under low water availability (<10% VWC). The relative growth rate (RGR) was significantly higher in *A. tortilis* (RGR=0.025) compared to *A. raddiana* (RGR= 0.011) during the drought period (14 days) (*t*_15_= -23.12, *p* < 0.001). In addition, the slope of the relation between daily growth and VWC was significantly (F_1,12 = _6.28, *p* < 0.001) steeper in *A. tortilis* than in *A. raddiana* (y = 94.539x - 1.147; y = 55.039x - 1.2294, respectively) revealing that *A. tortilis* plants used the available water better for gaining biomass. Therefore, the water-use efficiency of *A. tortilis* was significantly higher compared to *A. raddiana* (**Figure 4C**, F_1,9 = _25.61, *p* < 0.001). Moreover, to add a biochemical perspective, we measured the change of leaf sap osmolality in drought *vs*. control plants at the peak of drought (**Figure 4D**). Both species increased their osmolality during the drought period. However, the difference between drought and control plants was significantly larger in *A. tortilis* compared to *A. raddiana* (F_3,18 = _13.65, *p* < 0.001). i.e. in *A. tortilis* osmolality increased from 524 to 628 mmol kg^-1^ (in control *vs*. drought, respectively) while in *A. raddiana* osmolality increased from 544 to 591mmol kg^-1^ (in control *vs*. drought, respectively; **Figure S1**).

**Figure 5:**
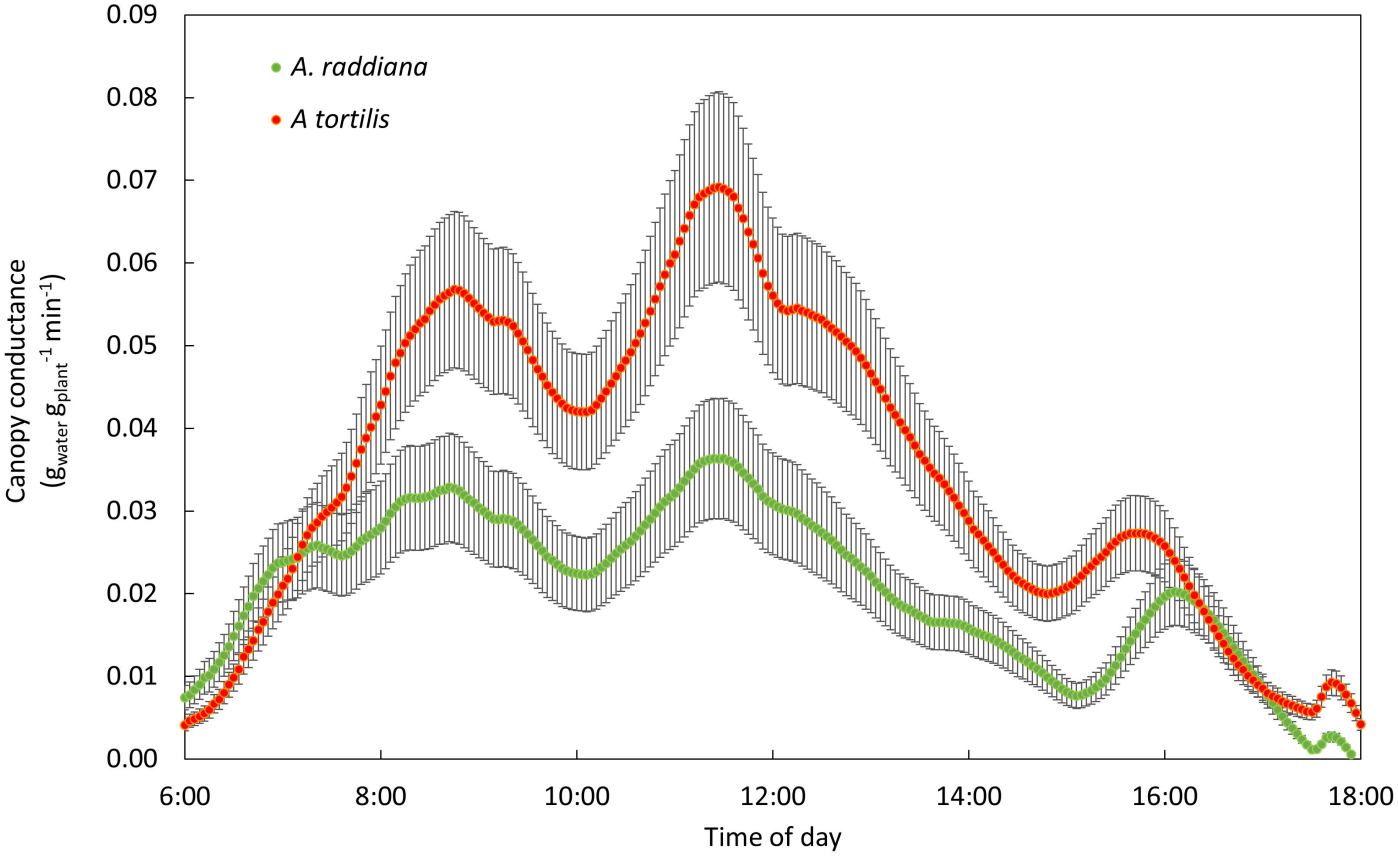
Recovery of *A. tortilis* (orange) and *A. raddiana* (green) in the first 24hr post 14 days of drought. Diurnal pattern of canopy conductance (n=5-7 average, SE error bars).

In the first 24 hr after re-irrigation of the plants in the drought treatment (simulating a high input of water typical of a flash flood event), we observed a significant difference between the two species in the ability to recover (**Figure 5**). *A. tortilis* seedlings transpired double the water amounts than *A. raddiana* at the daily peak (11:30) (0.06 ± 0.07, 0.03 ± 0.006 (g_water_/g_plant_/min)), respectively).

**Figure 6:**
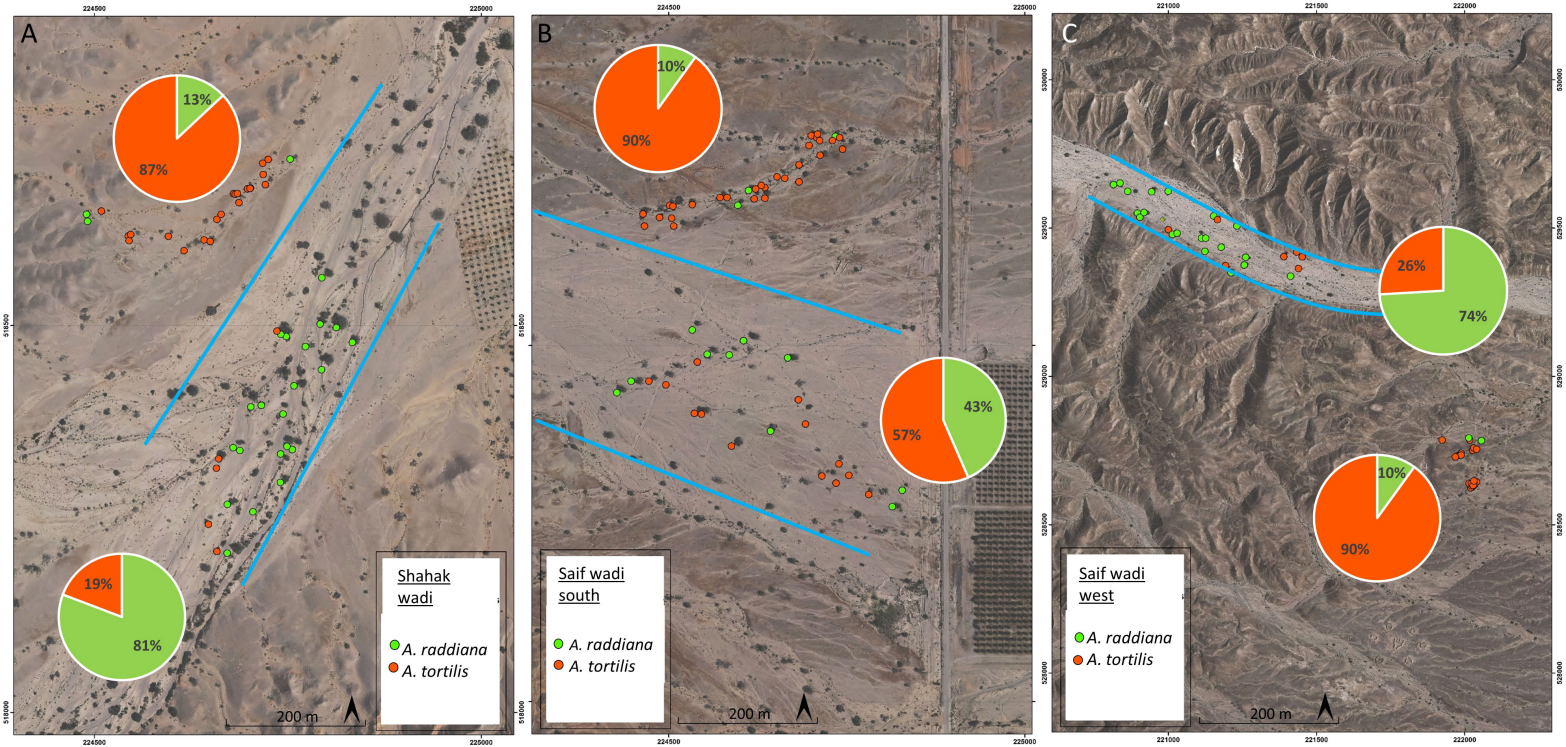
Orto-photos maps of tree species distribution in southern Arava, Israel. Shown are examples of different niche species distribution in three locations: **(A)** Shahak wadi (30.780597, 35.273345), **(B)** southern Saif wadi (30.836261N,35.259801E); **(C)** west Saif wadi (30.849811, 35.254353). Pie charts represent the abundance of *A. tortilis* (orange) and *A. raddiana* (green) in the main channel (in blue frame) and outside the main channel.

To put these results in a wider context, we also examined the distribution of the two species within the desert Wadi ecosystem. Three case studies from the hyper-arid desert in the Arava region showed that the abundance of *A. tortilis* in the main channel (trees within the blue frame in **Figure 6**) was low (33% of all trees in the main channel) compared to *A. raddiana* (64% of all trees). In contrast, outside the main channel (trees growing outside the blue frame) where less water flows and accumulates in the soil (Horton, 1945; Levick et al., 2008) there was a higher abundance of *A. tortilis* compared to *A. raddiana* (88% and 11% of all trees, respectively) (**Figure 6**).

## Discussion

### Understanding *Acacia* water-use strategy

A key question in plant science is why some plants do better than others in some environments, especially under drought. Here we performed an experiment in controlled conditions aiming to sort out the complex and intertwined responses found in the field, in order to understand the physiological response of Acacias to low water availability. A continuous and simultaneous tracking of whole-plant water-balance under different water availability conditions revealed that both *Acacia* species continued transpiring water even under low soil water availability (**Figures 3**, **4**). Maintaining transpiration at low water availabilities is unique in comparison to other plants, however, these values are in agreement to plants from savannas and dry shrub land regions (Fu et al., 2022). We also found that the canopy conductance of *A. tortilis* at the peak of drought and while recovering from a drought period was higher compared with *A. raddiana* (**Figures 4**, **5**). Although Acacia trees live in the most arid places on earth, with extremely low humidity both in the air and in the soil, here we showed a unique and non-trivial strategy of water use - a desert tree that keeps stomata open in very dry soil conditions (**Figure 3B**). Moreover, the diurnal trend of transpiration rate peaking around noontime remained the same even when the seedlings were exposed to extreme drought (**Figure 3**), similar to the pattern found in mature trees in the field (Uni et al., 2022). Hence, the plants that were exposed to low water availability did not stop growing (**Figures 2**, **4B**). Since stomatal closure is a key mechanism by which plants control their water status and avoid the negative effects of drought, our findings raise the question- why would a desert plant use such a water-spending strategy? Below we present two explanations: (1) the need for cooling the canopy and (2) the need to exploit all available water under conditions of high uncertainty.

### The desert tree dilemma – cooling *vs*. high water loss

In their natural habitats, *Acacia* trees cope with extremely high temperatures that exceed 40˚C at midday, every day throughout the summer, which lasts 4 months of the year (Goldreich and Karni, 2001; Winters et al., 2018). High temperatures of above 42°C cause denaturation of proteins, decrease the rate of chemical reactions and change the cell structural organization (Berry and Bjorkman, 1980). Opening of the stomata provides an evaporative cooling effect, cooling down the leaves when water is transpired (Crawford et al., 2012; Lapidot et al., 2019; Aparecido et al., 2020). However, opening of the stomata while VPD is extremely high (e.g., > 3.5 kPa) exposes the tree to the risk of excessive water loss (Chaves et al., 2003), loss of turgor (Bartlett et al., 2016) and xylem embolism (Wagner et al., 2022). Therefore, trees in hot desert environments face a dilemma, to transpire water in order to cool down (Lapidot et al., 2019), or to avoid high water loss by stomatal closure while taking a risk of overheating (Berry and Bjorkman, 1980; Crawford et al., 2012).

Measurements of Acacias’ canopy temperature (using thermal infra-red camera) in their natural hyper arid-habitat showed that mature trees cools *via* transpiration at the hottest hours (12:00) (Uni et al., 2022). Our results here show an anisohydric ‘water-spender’ strategy with a diurnal pattern of peak canopy conductance at noon (**Figure 3**) when temperatures are at their maximum, and we suggest, indirectly, that this ‘water- spender’ strategy might be a way to cope with high temperatures and survive where other tree species cannot. Other tree species, from semi-arid and Mediterranean forests, which cope with occasional high temperatures (maximum 40 ˚C) and a long dry season (with VPD of ~ 4 kPa), usually show a more water-conserving strategy, minimizing their activity during the dry season (and often also middays) to prevent water loss (Maseyk et al., 2008; Klein, 2014; Rog et al., 2021). For example, in a mixed dry Mediterranean forest, transpiration rates during summer were almost zero in *Pinus halepensis*, *Quercus calliprinos*, and *Cupressus sempervirens* (Rog et al., 2021). In addition, in a *P. halepensis* forest on the edge of the semi-arid region, reduction in stomatal conductance was observed with the seasonal increase in VPD (from 1 kPa to 4 kPa), resulting in transpiration rates of < 1 mmol m^-2^ s^-1^ in the summer months (Maseyk et al., 2008). Pine trees in this semi-arid region use convection to cool down their foliage (Muller et al., 2021), however this might be insufficient under higher temperature as in the Acacias’ hyper-arid natural habitat. On the other hand, when individual trees have higher water availability they can transpire more both in semi-arid pines (Tsamir et al., 2019) and the desert Acacias shown here. Moreover, in two similar controlled experiments on the same continuous and simultaneous tracking lysimeters system, *P. halepensis* seedlings showed a clear response to drought, responding with their stomata at relatively high soil water content of 39% leading to a reduction of the daily transpiration to a minimum (Houminer et al., 2022). Another hypothesis to explain the high stomata activity under drought might relate to the nitrogen fixation ability of the trees. Acacias associate with dinitrogen fixing rhizobia *via* root symbiosis (Sprent, 1995). Dinitrogen fixing bacteria in the root system can be a large sink for carbon, in turn increasing the demand for photosynthates, resulting in stomatal opening even during severe stress.

### Establishment of *Acacia* seedlings

We conducted our experiment on one-year-old Acacia seedlings, as a follow up experiment to a study on mature Acacia trees in their natural hyper-arid habitat (Uni et al., 2022). The seedlings studied here represent the most crucial stage of the establishment bottleneck, when seedling survival plays a critical role in the distribution and structure of the population of trees (Anderson et al., 2015; Morrison et al., 2019). A young Acacia seedling in the desert must cope with a low water supply that comes in high intensity, i.e., in pulses (flashfloods) together with a risk of erosion of the riverbed (Stavi et al., 2015). Thus, the young seedling has a short window of time to maximize carbon gain (growth) to ensure survival. The key factor to ensure survival in the desert is the ability to grow a long and wide root system that can reach deep water reservoirs (Castillo et al., 2002; Stave et al., 2005; Do et al., 2008; Sher et al., 2010; Winters et al., 2018) and creates mechanical stability to flashfloods (Stavi et al., 2015). Therefore, we suggest that the strategy of spending water in order to gain biomass helps the seedlings to grow deep root systems, thus providing an advantage for seedling survival and establishment in the desert. This is a “risk-taking” strategy, especially in extremely dry conditions that expose the Acacia seedlings to dehydration risk as they continue to transpire (**Figure 3B**) and grow (**Figure 4B**). Our findings are in accordance with Cory et al. (2022) that also describe an ‘an-isohydric’ approach used by *A. tortilis* seedlings from wetter environments (the Serengeti in Tanzania), indicating that this is not a local adaptation to the hyper-arid conditions in the Arava.

### Differential response to drought among co-occurring *A. raddiana* and *A. tortilis*

*A. tortilis* seedlings grew more than *A. raddiana* seedlings under low soil water content (**Figure 4B**) and exhibited a significantly higher water-use efficiency (biomass gain per water loss) (**Figure 4C**). The differences that we found between the two species in their physiological response to de- and re-hydrating soil suggest that *A. tortilis* may be more efficient in environments where the water pulses are more extreme (faster water movement, lower water accumulation).

Within their geographical distribution in the desert ecosystem, both species grow in ephemeral wadis (Zohary and Orshan, 1956; Stavi et al., 2015; Isaacson et al., 2017; Armoza-Zvuloni et al., 2021), however they differ in their preferential location within the wadi system (**Figure 6**). The main channel drains all the water in a certain catchment area, while outside the main channel wadis are characterized by fast water movement (Horton, 1945; Lange, 2005). Our results, which tested only the effect of SWC on Acacia canopy conductance and growth, may explain the observed distribution differences between the two species (**Figure 6**). *A. raddiana*, which showed a slower reaction to reduction and addition of water and lower WUE (**Figure 4C**) was found to be more abundant in the main channel, where there is larger amount of water in the soil and less fluctuations in water availability (Horton, 1945; Levick et al., 2008). On the other hand, *A. tortilis* utilize a more opportunistic strategy of water use (**Figures 2**, **4A**, **5**), and therefore has a significant advantage in growing outside the main channel, where water pulses are fast and extreme and accordingly, water availability in the soil is low and fluctuates greatly.

Further support for the risk-taking strategy in *A. tortilis* can be seen in the high increases of leaf osmolality in the drought treated compared to the control plants. It is well known that higher leaf osmolality is a biochemical adjustment to drought (Hsiao et al., 1976; Blum, 2017). Higher osmolality can also result from lower water content which increase the concentration of solutes in the sap (Taiz and Zeiger, 2006). Here we cannot disentangle the specific reason for the higher osmolality change in *A. tortilis*, but considering the spatial distribution of the species and even more so, the physiological response to drought (**Figure 4**), we suggest that osmoregulation is the appropriate explanation. In addition to the mechanisms discussed here, it should be noted that there are other processes involved in response to drought including increase in the concentration of abscisic acid, changes in Ca^2+^ and reactive oxygen species, and changes in the hydraulic conductance (Takahashi et al., 2020). There are also escape mechanisms such as reducing leaf area(Basu et al., 2016); however, the measurement of these responses were beyond the scope of this study.

The current study provides new insights regarding *Acacia* ecophysiology and their responses to low water availability. The research was performed on seedlings, providing whole-plant water-balance regulation under both wet and dry conditions in two species of Acacia from extreme desert co-habiting populations. Our findings provide a new understanding of how Acacia trees regulate the diurnal and total changes in canopy conductance, transpiration rates, and plant growth under extreme conditions. As desert vegetation is usually considered to take more conservative, risk-averse and desiccation-avoiding strategies, our results reveal a unique and non-trivial risk-taking strategy that potentially ensures the establishment of seedlings, with differences between the species that probably determine their distribution in the desert. Acacias thrive under extremely hot and dry conditions, which are predicted to be more prevalent in many places in the coming decades (Vicente-Serrano et al., 2010; Pachauri et al., 2014), suggesting these trees might survive future climate change and provide carbon sinks in a warmer and drier world.

## Data availability statement

The raw data supporting the conclusions of this article will be made available by the authors, without undue reservation.

## Author contributions

DU performed the experiment, the analysis, and the measurements under the guidance of ES, TK and GW. NS and RST created the acacia species distribution maps. DU wrote the paper with ES, TK and GW. All authors contributed to the article and approved the submitted version.

## Supplementary material

Supplementary Figure 1 Leaf osmolality (mmol/kg) among the four groups of the experiment as measured at the beginning of the experiment (23.7.20) **(A)**, at the peak of drought (31.8.20) **(B)** and at the recovery phase (6.9.20) **(C)**. Significant higher values of osmolality were measured in *A. tortilis* at the drought treatment.
