# Supplementary figures and images for: Responses of two Acacia species to drought suggest different water-use strategies, reflecting their topographic distribution
Source: Front Plant Sci. 2023 Jun 5;14:1154223. doi: 10.3389/fpls.2023.1154223 (PMC10277743; doi:10.3389/fpls.2023.1154223)

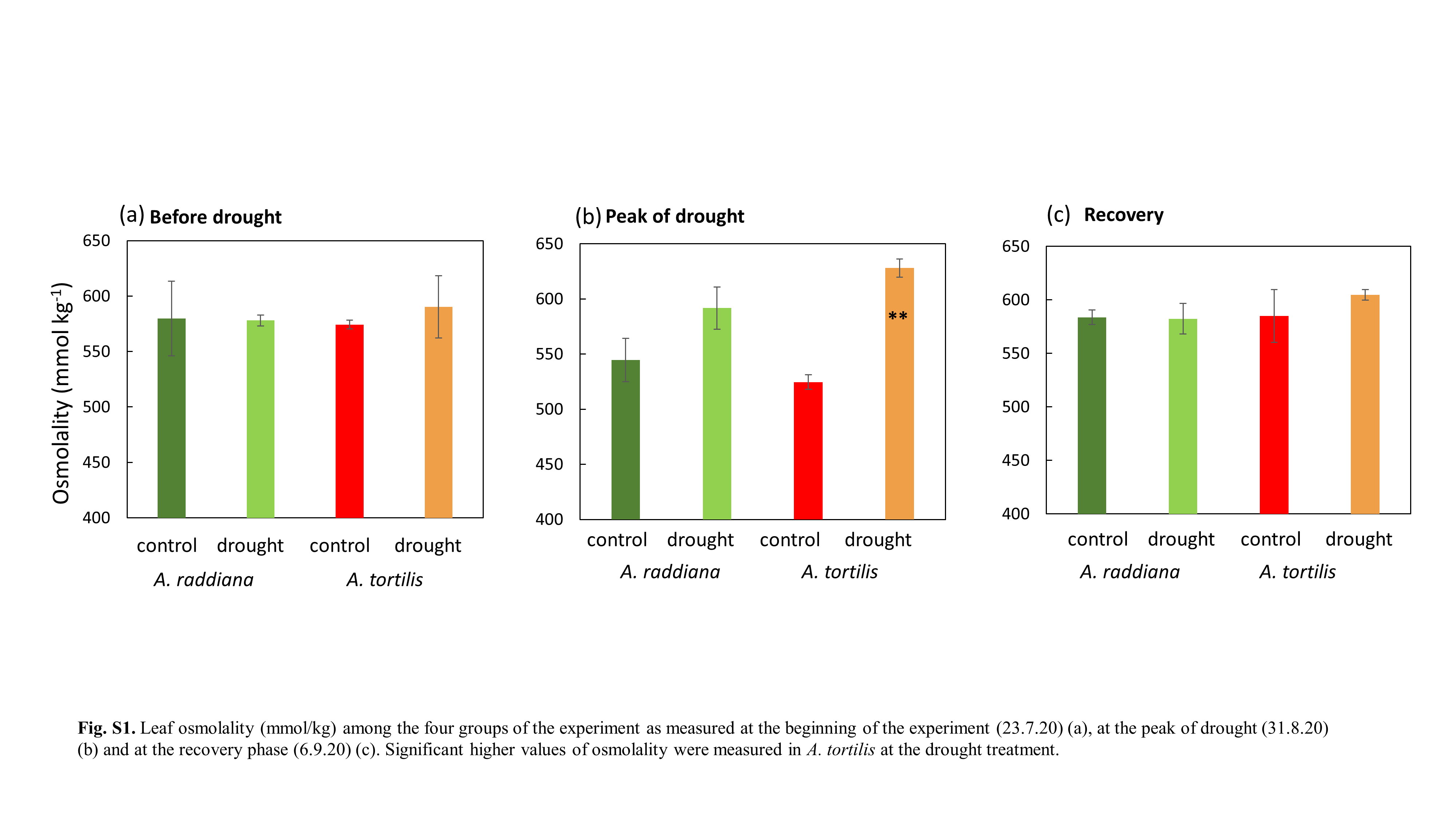

Supplement: Supplementary Figure 1 — Leaf osmolality (mmol/kg) among the four groups of the experiment as measured at the beginning of the experiment (23.7.20) (A), at the peak of drought (31.8.20) (B) and at the recovery phase (6.9.20) (C). Significant higher values of osmolality were measured in A. tortilis at the drought treatment. [file Image_1.tif]
